# Bortezomib-Induced Pulmonary Toxicity: A Case Report and Review of Literature

**DOI:** 10.1155/2018/2913124

**Published:** 2018-11-25

**Authors:** Prakash Kharel, Deekchha Uprety, Abhinav B. Chandra, Yirui Hu, Anuradha Avinash Belur, Ajay Dhakal

**Affiliations:** ^1^Department of Hospital Medicine, Geisinger Medical Center, 100 N Academy Ave., Danville, PA 17821, USA; ^2^Renal Division, Brigham and Women's Hospital, Boston, MA, USA; ^3^Yuma Regional Medical Center, Yuma, AZ, USA; ^4^Center for Health Research, Geisinger Medical Center, 100 N Academy Ave., Danville, PA 17821, USA; ^5^Multi Care Regional Cancer Center, Auburn, WA, USA; ^6^Department of Medicine, Wilmot Cancer Institute, University of Rochester, Rochester, NY, USA

## Abstract

Bortezomib, a proteasome inhibitor, is an established therapy against multiple myeloma. Bortezomib-induced lung injury, although not appreciated during the introductory time of the medication, has now been highlighted in multiple case reports. The objective of this study is to report a case of bortezomib-induced lung injury, review current literature, and perform exploratory analysis.

## 1. Introduction

The proteasome inhibitor bortezomib (BTZ) suppresses (NF)-*κ*B, which is an important mediator of inflammation. BTZ is an established therapy against multiple myeloma (MM). Most of the adverse effects associated with bortezomib are mild to moderate and are generally manageable, including gastrointestinal symptoms, peripheral neuropathy, and thrombocytopenia [1]. BTZ-induced lung injury (BLI), although not appreciated during the introductory time of the medication, has now been highlighted in multiple case reports [1–21].

Underlying mechanism of BLI is unclear but a proposed hypothesis is that BTZ withdrawal leads to rebound activation of (NF)-*κ*B causing inflammatory changes in the lungs. This explains the rapid improvement of BLI with steroid therapy reported in several cases. But, there are a number of cases reported, which did not improve with steroid. Alternate hypothesis is that BTZ metabolites accumulate in the lung tissue causing direct lung injury.

Some anecdotes claim that genetic predisposition (especially Japanese population) and history of prior stem cell transplant (SCT) might be risk factors for BLI [1, 9, 21]. The objective of this study is to report a case of BLI, review current literature, and determine the predictors of mortality in BLI based on the analysis of the data from the cases reported.

## 2. Case Report

**Figure 1:**
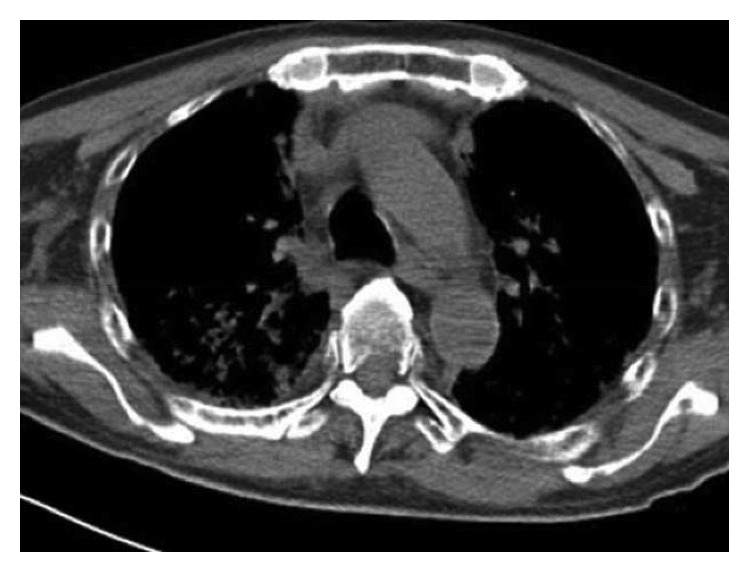
CT chest showing new interval appearance of bilateral perihilar ground-glass opacities and peribronchial and interstitial thickening predominantly in the upper lobes.

**Figure 2:**
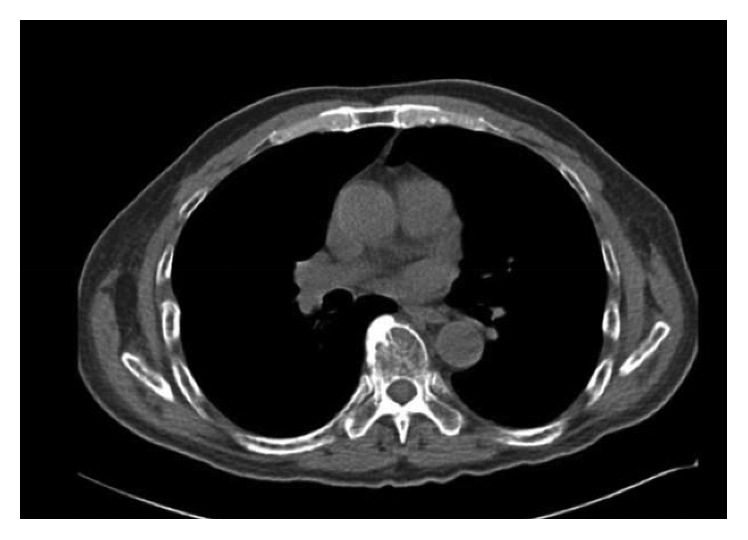
Prior CT chest for comparison.

**Figure 3:**
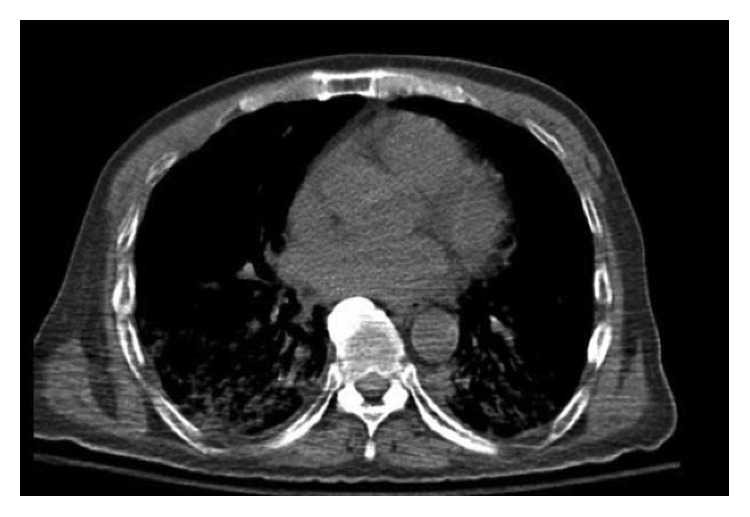
A repeat chest CT showed resolution of previously well-defined areas of perihilar ground-glass opacities but development of hazy areas of ground-glass opacification throughout both lungs with more confluent abnormalities in bilateral lower lobes.

A 64-year-old male with chronic low back pain presented to the emergency department with a new onset, severe mid back pain radiating to bilateral shoulders. CT scan, performed to rule out aortic dissection, demonstrated multiple lytic lesions throughout the bony skeleton and a compression fracture at T7 vertebral body with epidural extension of the soft tissue. A bone survey confirmed CT scan findings, and subsequent bone marrow biopsy confirmed the diagnosis of MM. He received radiation therapy to the thoracic spine and completed 2 cycles of CyBorD regimen (cyclophosphamide 300 mg/m^2^ by mouth, BTZ 1.5 mg/m^2^ subcutaneous, and dexamethasone 40 mg by mouth each on days 1, 8, 15, and 22). Three days after the completion of the second cycle, he was admitted to hospital with respiratory distress. CT chest (Figure 1) showed new interval appearance of bilateral perihilar ground-glass opacities and peribronchial and interstitial thickening predominantly in the upper lobes not seen in prior scan (Figure 2). There were no other signs or symptoms of pneumonia such as leukocytosis, fever, or cough. After some benefit from oral prednisone, he was discharged with a tapering dose of the same. Unfortunately, the patient was readmitted with worsening respiratory distress 4 days later. A repeat CT scan of the chest showed resolution of previously well-defined areas of perihilar ground-glass opacities but development of hazy areas of ground-glass opacification throughout both lungs with more confluent abnormalities in bilateral lower lobes (Figure 3). The patient was treated with high-dose methylprednisone and noninvasive positive pressure ventilation without any improvement. The family requested do-not-resuscitate and do-not-intubate status. The patient died on the 10^th^ day of the admission.

## 3. Methodology

We have reported a case and performed a review of current literature on BLI and statistical analysis of available data. Extensive PubMed search was performed to screen the literature using the search words “bortezomib” and “pulmonary injury,” “pulmonary toxicity,” “lung injury,” “lung toxicity,” “pneumonitis,” “pulmonary side effects,” “lung side effects,” “shortness of breath,” “breathlessness,” “dyspnea,” “ARDS,” or “respiratory failure.” BLI was defined as new onset of respiratory symptoms with new radiological finding without evidence of infection and other causes of respiratory symptoms including cardiac causes and other drug-related adverse effects. Respiratory symptoms included fever, dyspnea, cough, breathlessness, or respiratory failure. Radiological findings included any pulmonary infiltrate, pleural effusion, pulmonary edema, ground-glass opacity, diffuse alveolar hemorrhage, reticular shadowing, or features of pulmonary hypertension and interstitial pneumonitis. Eligible cases from the literature were included in the review.

## 4. Results

An extensive PubMed search yielded 35 events of BLI, 5 of which occurred with readministration of BTZ after resolution of initial BLI. The mean (standard deviation SD, minimum, maximum) age is 61.14 years (9.93, 31, 78) and 65.71% were male. 14.29% had current or past smoking history, 17.24% had history of lung disease, and 31.43% had prior SCT for MM. 14.29% received steroid just prior to or along with bortezomib. 14.29% were receiving concurrent chemotherapy. The mean total BTZ dose was 4.41 mg (2.75, 1, 9), mean duration from the first dose to the onset of BLI was 20.59 days (16.59, 0.5, 60), and the mean duration from the last dose to onset of BLI was 3.08 days (2.77, 0, 10). 37.14% of BLIs were fatal.

The *t*-test showed no significant difference in mean age, mean number of BTZ doses, and mean duration from the 1^st^ dose of BTZ to the onset of BLI between deceased and survivors. But, the difference in mean duration (SD, minimum, maximum) from the last dose of BTZ to the onset of BLI between deceased (1.59 days (SD, 0, 5)) and survivors (3.95 days (SD, 0, 10)) were found to be statistically significant (*p*=0.009) Table 1. 45.45% deceased had prior SCT compared to 11.11% deceased who had no stem cell transplant (*p*=0.0360). No significant association could be found between the outcome of BLI and various factors like sex, previous lung diseases, history of smoking, concurrent/prior steroids, and treatment of BLI with steroids Table 2.

## 5. Discussion

Most of the adverse effects associated with bortezomib are mild to moderate and are generally manageable, including gastrointestinal symptoms, peripheral neuropathy, and thrombocytopenia [1]. BLI is a serious yet underappreciated adverse effect of BTZ associated with a high mortality rate of 37.14% as suggested by our analysis of reported cases available. Some initial case reports suggested a mortality rate as high as 50% [1]. In one phase IV clinical study of 666 patients who had received bortezomib published by Narimatsu et al., 3.6% of pulmonary complications and 0.5% of total deaths were attributed to these complications [22]. Some studies have shown that genetic predisposition to BLI, especially with Japanese population, and history of prior SCT might be risk factors for BLI [1, 9, 21]. Some cases responded very well to steroids [11], while others did not. Our patient responded to steroid to some extent and was discharged. He later presented with worsening pulmonary symptoms and died. It is important to establish the dose and duration of steroid for the patients who respond to therapy. For others who do not respond to steroid, there is a need to identify other mechanisms of BLI, so that appropriate treatment may be directed. Clinicians should have a high degree of suspicion for BLI, as it can be misjudged as other more common respiratory conditions. Appropriate supportive management along with steroids and immediate withdrawal of BTZ should be the primary management strategy.

Our study showed that fatal BLIs were associated with early onset after the last dose of BTZ (with mean 1.59 days vs. 3.95 days among nonfatal BLI) and with history of prior SCT. No significant association could be found between the outcome of BLI and various factors like sex, previous lung diseases, history of smoking, concurrent/prior steroid intake, concurrent steroid as a part of chemotherapy regimens, and steroid administered to treat BLI. Small sample size, retrospective nature of the study, and analysis of composite data from the reported cases in the PubMed are major limitations of the study.

Carfilzomib (CFZ) is an irreversible, second-generation selective proteasome inhibitor that is used to treat relapsed or refractory MM. In July 2012, the FDA granted approval of carfilzomib for the treatment of patients with multiple myeloma who have received at least two prior therapies including bortezomib and an immunomodulatory drug and who have shown disease progression while on therapy or within 60 days of completion of the last therapy. In the ASPIRE study (a phase III trial of CFZ plus lenalidomide (R) and dexamethasone (D) vs. RD only in relapsed MM) and the ENDEAVOR study (a phase III trial of CFZ plus D vs. bortezomib plus D in relapsed MM), there was slightly higher incidence of all-grade dyspnea in the CFZ arm compared to respective controls (29 versus 13 percent) and cough (25 versus 14 percent), but there were no BLI-like noninfectious progressive lung injuries reported [23, 24]. According to FDA-approved manufacturer's prescribing information, carfilzomib was associated with development of pulmonary hypertension in 2% of patients [25]. Severe pulmonary hypertension (grade ≥ 3) was in less than 1%.

Based on our literature review, only 5 case reports of CFZ-associated noninfectious progressive lung injury have been identified. In one of the reported cases, the patient presented with dyspnea on exertion, fatigue, and a dry cough after the second dose of CFZ which got worse with subsequent dose until CFZ was discontinued. A CT chest revealed diffuse bilateral ground-glass micronodular opacities and increased reticular markings. The pulmonary function test showed a severe restrictive ventilatory defect, with severely reduced diffusion capacity and desaturation to 84% on exertion. Symptoms and CT scan finding improved after oral prednisone [26]. In other case report, the patient developed an acute respiratory distress syndrome requiring mechanical ventilation. After a week, the patient developed diffuse alveolar hemorrhage and despite aggressive supportive care, the patient died after three weeks [27]. The third case report was also about fatal pulmonary hemorrhage after CFZ. The patient had developed acute dyspnea within 2-3 hours of first dose of CFZ, along with infiltrates on chest X-ray. Symptoms resolved after corticosteroid and bronchodilators along with resolution of X-ray finding next day. The patient was subsequently treated five scheduled doses without any problem. After about a month, when second cycle of CFZ was given, the patient again developed acute dyspnea, with no resolution of symptoms. The patient later died of pulmonary hemorrhage despite aggressive therapy [28]. Recent case series reported one case of pulmonary hypertension after CFZ and other case of dyspnea with lung disease with pulmonary function test showing significantly decreased unadjusted diffusion capacity of the lung [29]. With increasing use of CFZ, more information may emerge regarding the CFZ-associated lung injuries in future.

## 6. Conclusion

BLI is a serious condition with a mortality rate of 37.14%. Early onset of BLI from the last dose of BTZ and prior SCT can be the predictors of mortality. Further studies are needed to confirm these findings. Limited data have emerged on the possible association of CFZ with various lung injuries. Clinicians should be mindful of this potentially fatal adverse effect of proteasome inhibitors like BTZ or CFZ.

## Figures and Tables

**Table 2:** Comparison of age, total BTZ dose, time from first dose of BTZ, and time from last dose of BTZ between those who survived and those who died (*N*=35).

Characteristics	Fatal cases (*N*=xx)	Nonfatal cases (*N*=yy)	*p* value^*∗*^
	Mean (SD)	Mean (SD)	
Age	60.54 (13)	61.50 (7.91)	0.812
Total BTZ	4.92 (2.94)	4.10 (2.65)	0.439
Time from 1st dose of BTZ	22.96 (18.03)	19.24 (15.84)	0.558
Time from last dose of BTZ	1.59 (1.66)	3.95 (2.95)	0.009

^*∗*^ *t*-test.

**Table 1:** Outcome based on individual categorical variables: sex, new/relapsed/refractory type, prior SCT, smoking, previous lung injury, steroid just prior to BTZ, and concurrent chemotherapy (*N*=35).

Variable		Outcome		*p* value^*∗*^
		Death (%)	Resolution of BLI (%)	
Sex	Female	6 (50)	6 (50)	0.2555
	Male	7 (30)	16 (70)

Type of BLI	New	3 (33)	6 (67)	0.8450
	Relapsed	7 (37)	12 (63)
	Refractory	2 (50)	2 (50)

Prior SCT	No	2 (11)	16 (89)	0.0360
	Yes	5 (45)	6 (55)

Smoking	No	6 (35)	11 (65)	0.1193
	Yes	0 (0)	5 (100)

Previous lung injuries	No	10 (50)	10 (50)	0.4726
	Yes	2 (33)	4 (67)	

Prior or concurrent steroids	No	10 (34)	19 (66)	0.8116
	Yes	2 (40)	3 (60)

Concurrent chemotherapy	No	12 (41)	17 (59)	0.3636
	Yes	1 (20)	4 (80)

^*∗*^Chi-square test.

## Abbreviations

BTZ: Bortezomib
BLI: BTZ-induced lung injury
SCT: Stem cell transplant
SD: Standard deviation.
